# A serum metabolic biomarker panel for early rheumatoid arthritis

**DOI:** 10.3389/fimmu.2023.1253913

**Published:** 2023-09-01

**Authors:** Samantha Rodríguez-Muguruza, Antonio Altuna-Coy, Verónica Arreaza-Gil, Marina Mendieta-Homs, Sonia Castro-Oreiro, Maria José Poveda-Elices, Nuria del Castillo-Piñol, Ramon Fontova-Garrofé, Matilde R. Chacón

**Affiliations:** ^1^ Disease Biomarkers and Molecular Mechanisms Group, Institut d'Investigació Sanitària Pere Virgili (IISPV), Universitat Rovira i Virgili, Tarragona, Spain; ^2^ Rheumatology Department, Joan XXIII University Hospital, Tarragona, Spain

**Keywords:** early rheumatoid arthritis, metabolomics, serum, diagnostic, biomarkers 2

## Abstract

**Objective:**

There is an urgent need for novel biomarkers to improve the early diagnosis of rheumatoid arthritis (ERA). Current serum biomarkers used in the management of ERA, including rheumatoid factor and anti-cyclic citrullinated peptide (ACPA), show limited specificity and sensitivity. Here, we used metabolomics to uncover new serum biomarkers of ERA.

**Methods:**

We applied an untargeted metabolomics approach including gas chromatography time-of-flight mass spectrometry in serum samples from an ERA cohort (n=32) and healthy controls (n=19). Metabolite set enrichment analysis was performed to explore potentially important biological pathways. Partial least squares discriminant analysis and variable importance in projection analysis were performed to construct an ERA biomarker panel.

**Results:**

Significant differences in the content of 11/81 serum metabolites were identified in patients with ERA. Receiver operating characteristic (ROC) analysis showed that a panel of only three metabolites (glyceric acid, lactic acid, and 3-hydroxisovaleric acid) could correctly classify 96.7% of patients with ERA, with an area under the ROC curve of 0.963 and with 94.4% specificity and 93.5% sensitivity, outperforming ACPA-based diagnosis by 2.9% and, thus, improving the preclinical detection of ERA. Aminoacyl-tRNA biosynthesis and serine, glycine, and phenylalanine metabolism were the most significant dysregulated pathways in patients with ERA.

**Conclusion:**

A metabolomics serum-based biomarker panel composed of glyceric acid, lactic acid, and 3-hydroxisovaleric acid offers potential for the early clinical diagnosis of RA.

## 1 Introduction

Rheumatoid arthritis (RA) is the most frequent inflammatory rheumatic disease, with a prevalence of 0.5–1% in the adult population; the disease increases in incidence with age and affects women more than men (1). The etiology of RA remains unknown, but multiple mechanisms appear to be involved (2). Given the heterogeneity of clinical manifestations and the variability of therapeutic response, the early diagnosis of RA is essential to guide therapy and prevent disease progression.

Metabolomics is a powerful technology to explore changes in the abundance of metabolites within a biological system and can provide valuable insights into the metabolic pathways that are perturbed in different diseases, including RA (3).

Metabolite levels in the serum and urine of patients with RA can exhibit significant changes compared with healthy individuals or individuals with other conditions. These changes are typically associated with the underlying inflammatory processes, immune dysregulation, and oxidative stress commonly observed in patients with RA (4). Although the metabolic changes can be complex and vary between patients, there are some key metabolites and common metabolic pathways; for example, amino acid metabolism has been reported to be altered in patients with RA, who present with elevated levels of pro-inflammatory amino acids such as arginine, citrulline, and ornithine and lower levels of anti-inflammatory amino acids such as tryptophan (4).

Dysregulation of lipid metabolism is also a common trait in RA, and elevated levels of cholesterol and triglycerides have been reported in the serum of patients (5). Indeed, abnormal lipid profiles, including changes in phospholipids and fatty acids, have been detected in both serum and urine (6). Moreover, changes in energy metabolism have been described in both the serum and urine of patients with RA, including alterations in the levels of the energy substrates glucose and lactate (7), higher levels of the oxidative stress marker malondialdehyde, and an increase in advanced oxidation protein products, among others (8, 7).

Alterations in metabolite levels have also been found to reflect different therapeutic effects in patients with early RA (ERA). Metabolites such as uric acid, taurine, methionine, glycine, histidine, and hypoxanthine have been found to be elevated in the serum of patients with ERA after responding to methotrexate treatment with respect to patients for whom the drug was not effective (9). Contrastingly, uracil, trimethylamine N-oxide, oxoglutarate, aspartate, and tryptophan levels were decreased in the same studied cohorts (10).

Monitoring metabolic changes in ERA may aid in understanding disease progression and treatment response. Although several biofluid metabolomic biomarkers have been reported as useful in the stratification of patient-response prediction to a specific therapy, very few studies have investigated novel biomarkers to improve the early diagnosis of RA. Accordingly, in the present study, we performed serum metabolite profiling in a well-characterized and representative cohort of patients with ERA naïve to treatment *versus* a matched control cohort to identify a metabolic-related panel to detect ERA. Putative signaling pathways related to dysregulated metabolites were also analyzed to better understand the onset of the disease.

## 2 Materials and methods

### 2.1 Study population

This is an observational case-control study. Patients were recruited at the Joan XXIII University Hospital in Tarragona (Spain). Patients (n=32) were classified as having ERA according to ACR/EULAR 2010 criteria, with a symptoms duration of ≤6 months and not receiving treatment with glucocorticoids or synthetic or biological disease-modifying drugs. Control subjects (n=19) were individuals with no relevant medical history. Groups were matched for sex and age. The study was performed according to the provisions of the Declaration of Helsinki, was approved by the local ethics committee, and adhered to current legal regulations (Bio-medical Research Law 14/2007, Royal Decree of Biobanks 1716/2011, Organic Law15/1999 of September 13 Protection of Personal Data). All methods were approved by the Ethical Committee for Clinical Research (CEIM) of the Pere Virgili Research Institute (Ref. CEIM: 047/2021). All participants gave written informed consent. The following exclusion criteria were applied: patients unwilling or unable to provide informed consent; patients having a diagnosis of any systemic inflammatory condition other than RA, such as (but not limited to) juvenile chronic arthritis, spondyloarthropathy, Crohn’s disease, ulcerative colitis, psoriatic arthritis, active vasculitis, or gout (participants with secondary Sjogren’s syndrome were not excluded); history or presence of cardiovascular, respiratory, hepatic, gastrointestinal, endocrine, hematological, neurological, or neuropsychiatric disorders; or any other serious and/or unstable illness that, in the opinion of the investigator, could constitute a risk when taking investigational samples or could interfere with the interpretation of data.

### 2.2 Serum sample collection and analytical methods

Fasted blood was extracted, and glucose, cholesterol, triglyceride, high-density lipoprotein cholesterol, and hepatic and renal profiling were performed using the standard methods. C-reactive protein (CRP), rheumatoid factor, and anti-cyclic citrullinated peptide (ACPA) levels were determined using standard enzymatic methods.

### 2.3 Untargeted metabolomic analysis of serum samples

The metabolomic analysis was performed at the Center for Omic Sciences (Eurecat, Reus, Spain). Samples (100 μL serum) were aliquoted into 1.5 mL tubes and mixed with 400 μL of a solution of methanol: water (8:2) containing internal standards. Samples were vortexed and centrifuged for 5 min at 15000 rpm and at 4°C. Supernatants (200 μL) were transferred to new tubes and evaporated in a SpeedVac at 45°C. Samples were then reconstituted with 30 μL of methoxyamine and incubated for 90 min at 37°C, after which time they were silylated with the addition of 45 μL of MSTFA + 1% TMCS at room temperature for 60 min.

Gas chromatography time-of-flight mass spectrometry (GC-TOF-MS) separation was performed with helium (purity >99.999%) as a carrier gas at a constant flow of 1.1 mL/min. The initial GC oven temperature was 60°C; 1 min after injection, the GC oven temperature was increased by 10°C/min to 320°C and held for 10 min at 320°C. The samples were injected with split mode 1:20 at an injection temperature of 250°C. Detection was achieved using MS in electron ionization (70 eV) mode and full-scan monitoring mode (m/z 50–600) with an acquisition rate of 5 spectra/s. The temperature of the ion source was set at 250°C with the quadrupole at 200°C.

Organic acids were identified and semi-quantified using their pure analytical standards. Specifically, ions were selected and used for quantification based on their impact electron spectra (70 eV) and the main specific ions described in the recorded spectra library Fiehn (Agilent-Technologies Inc. USA).

### 2.4 Statistical data analysis

The sample size was estimated using G*Power 3.1.9.7 (https://www.psychologie.hhu.de/arbeitsgruppen/allgemeine-psychologie-und-arbeitspsychologie) (11). A 2-fold change between groups and similar group variances, with an average power of >80% and an alpha error of 0.1 and an effect size of 1 was assumed; a minimum of 19 patients in each group were needed.

Analysis of the differences between the qualitative clinical data between study groups was carried out using the Chi-square test. The Shapiro-Wilk test was used to assess the normality of clinical, anthropometric, and metabolomics variables. The non-parametric Mann-Whitney U test was used to analyze differences in the anthropometric, analytical data and metabolomic data between controls and patients with ERA. Data were expressed as median (25th percentile, 75th percentile range). To identify metabolites that could discriminate between controls and ERA, partial least square regression discriminant analysis (PLS-DA) was used. Variable importance projection (VIP) scores were examined to select the best-discriminating variables, whereby a VIP score of >1 was considered important in the discrimination. Binary logistic regressions and receiver operating characteristic (ROC) curve analysis were performed to identify the best discriminating metabolic model between the groups.

MetaboAnalyst 5.0 software (https://www.metaboanalyst.ca), a package based on R software environment (https://github.com/xia-lab/MetaboAnalystR), was used to obtain a better understanding of which metabolic pathways were enriched and were associated with dysregulated metabolites in the Kyoto Encyclopedia of Genes and Genomes (KEGG) metabolite library.

SPSS statistical software (Statistics 26.0, IBM, Madrid, Spain) was used to perform all the statistical analyses, and GraphPad Prism v9 (GraphPad Software Inc., San Diego, CA) was used to generate box plots. A p-value of ≤ 0.05 was considered significant in all analyses.

## 3 Results

### 3.1 Metabolomic profiling

**Figure 1:**
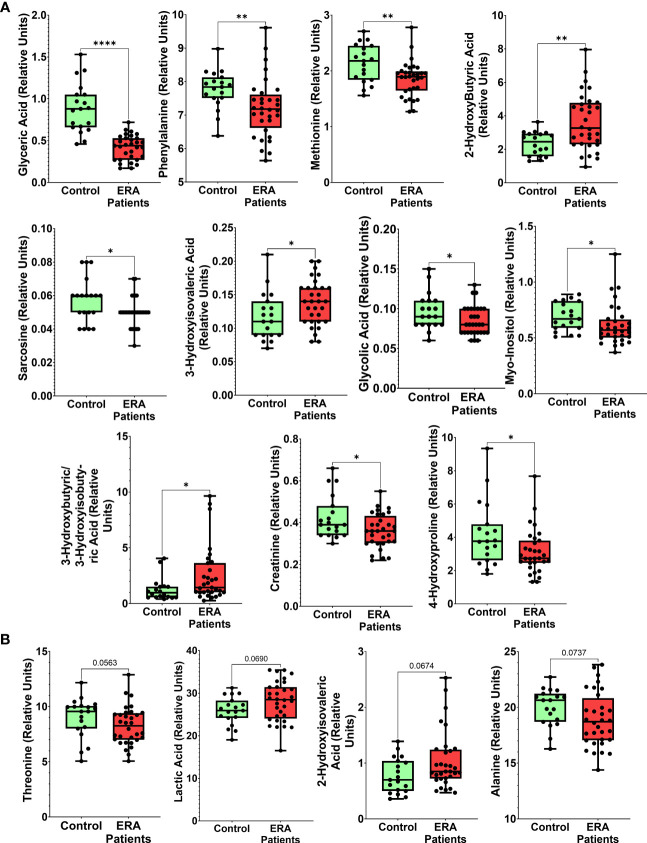
Metabolites differentially altered in serum from patients with ERA. Box plots of relative abundance of the 15 discriminatory metabolites analyzed in serum: **(A)** Significantly different metabolites between ERA and healthy controls, **(B)** Dysregulated metabolites with no significant differences. Results are expressed as median (25th percentile, 75th percentile range). Statistical differences between groups are indicated: * p < 0.05; ** p < 0.01; ***p < 0.001, ****p<0.0001.

We performed untargeted metabolomic profiling of serum from 32 patients with ERA and 19 healthy controls. The baseline characteristics of the participants are described in **Table 1**. A total of 81 individual metabolites were detected by GC-TOF-MS (**Supplementary Material**, **Table S1**), but the concentrations of only 11 were significantly different between ERA and control samples: methionine, 2-hydroxybutyric acid, and 3-hydroxyisovaleric acid were higher in ERA serum than in control serum, whereas 3-hydroxybutyric acid/3-hydroxyisobutyric acid, glycolic acid, sarcosine, glyceric acid, 4-hydroxyproline, creatinine, phenylalanine, and myo-inositol were lower (**Table 2**, **Figure 1A**). The levels of four metabolites (threonine, lactic acid, 2-hydroxyisovaleric acid, and alanine) were also different between groups but did not reach statistical significance (**Table 2**, **Figure 1B**).

**Table 1:** Baseline characteristics of the studied cohorts.

	Control (n=19)	ERA (n=32)	
Variables	Median (IQR)	Median (IQR)	*P* value
**Age (years)**	48.00 (45.00, 52.00)	50.50 (42.50, 55.75)	0.301
**BMI (kg/m^2^)**	25.61 (24.72, 28.41)	27.30 (23.90, 30.76)	0.681
**Sex (F/M)**	14/5	22/10	0.772
**Smoking (Yes/No)**	8/11	16/16	0.761
**Glucose (mM)**	5.24 (4.38, 5.52)	4.80 (4.50, 5.24)	0.355
**Creatinine (mM)**	64.80 (59.70, 71.80)	56.14 (50.83, 70.50)	0.067
**Total cholesterol (mM)**	5.11 (4.21, 5.86)	4.84 (4.22, 5.44)	0.299
**HDL-cholesterol (mM)**	1.28 (0.93, 1.40)	1.13 (0.91, 1.32)	0.554
**Triglycerides (mM)**	1.11 (0.82, 1.47)	1.10 (0.80, 1.62)	0.772
**GGT (μkat/L)**	0.27 (0.21, 0.42)	0.26 (0.21, 0.39)	0.605
**C-reactive protein (mg/L)**	1.13 (0.72, 1.71)	6.80 (3.70, 20.00)	<0.001
**Anti-ACPA (U/ml)**	1.41 (1.16, 1.52)	116.50 (45.55, 317.50)	<0.001
**RF (UI/mL)**	ND	158.50 (41.25, 309.25)	-

BMI, body mass index; HDL, high-density lipoprotein; GGT, gamma-glutamyl transferase; CRP; C-reactive protein, ACPA, anti-citrullinated protein/peptide antibody; RF, rheumatoid factor; ND, not determined; F, female; M, male; mM, millimolar; L, liter, μkat; microkatal, IQR; interquartile range.

**Table 2:** Metabolite levels in the serum of patients with ERA and controls.

Metabolites (Relative Units)	Control (n=19)	Early RA (n=32)	*p-value*
	Median (IRQ)	Median (IQR)	
**Lactic Acid**	25.90 (24.16, 28.24)	28.46 (24.04, 31.44)	0.07
**Glycolic Acid**	0.09 (0.08, 0.11)	0.08 (0.07, 0.10)	0.028
**Alanine**	20.64 (18.70, 21.22)	18.71 (17.05, 20.83)	0.073
**2-Hydroxybutyric Acid**	2.45 (1.59, 2.94)	3.27 (2.30, 4.77)	0.008
**Sarcosine**	0.06 (0.05, 0.06)	0.05 (0.05, 0.05)	0.017
**3-Hydroxybutyric Acid/3-Hydroxyisobutyric Acid**	0.98 (0.57, 1.52)	1.44 (1.02, 3.64)	0.034
**2-Hydroxyisovaleric Acid**	0.70 (0.50, 1.04)	0.85 (0.72, 1.25)	0.073
**3-Hydroxyisovaleric Acid**	0.11 (0.09, 0.14)	0.14 (0.11, 0.16)	0.023
**Glyceric Acid**	0.88 (0.66, 1.05)	0.44 (0.27, 0.53)	<0.001
**Threonine**	9.56 (8.01, 10.06)	8.25(6.99, 9.40)	0.059
**Methionine**	2.18 (1.84, 2.45)	1.89 (1.64, 1.99)	0.008
**4-Hydroxyproline**	3.76 (2.62, 4.78)	2.74 (2.46, 3.80)	0.039
**Creatinine**	0.39 (0.34, 0.48)	0.36 (0.31, 0.43)	0.039
**Phenylalanine**	7.84 (7.51, 8.13)	7.18 (6.62, 7.62)	0.004
**Myo-Inositol**	0.67 (0.59, 0.83)	0.57 (0.50, 0.66)	0.029

### 3.2 Analysis of serum signatures for diagnosis of early RA

To identify the most relevant metabolites that would allow us to correctly identify ERA based on the serum metabolomic profile, we performed a PLS-DA data analysis using data sets of the aforementioned 15 deregulated metabolites. The score plot of the PLS-DA model showed a partial separation between patients and controls (**Supplementary Material**, **Figure S1A**), but the model overfitting, measured as the Q2/R2 ratio (R2—how well the model predicts the calibration of variables, and Q2—how well the model predicts ERA) was 0.38, indicating a moderate level of predictive accuracy (**Supplementary Material**, **Figure S1B**). A model is considered predictive when the Q2/R2 ratio is greater than 0.5 (11). A heat map (**Supplementary Material**, **Figure S1C**) based on the 15 metabolic features showed a trend for ERA features to cluster separately.

**Figure 2:**
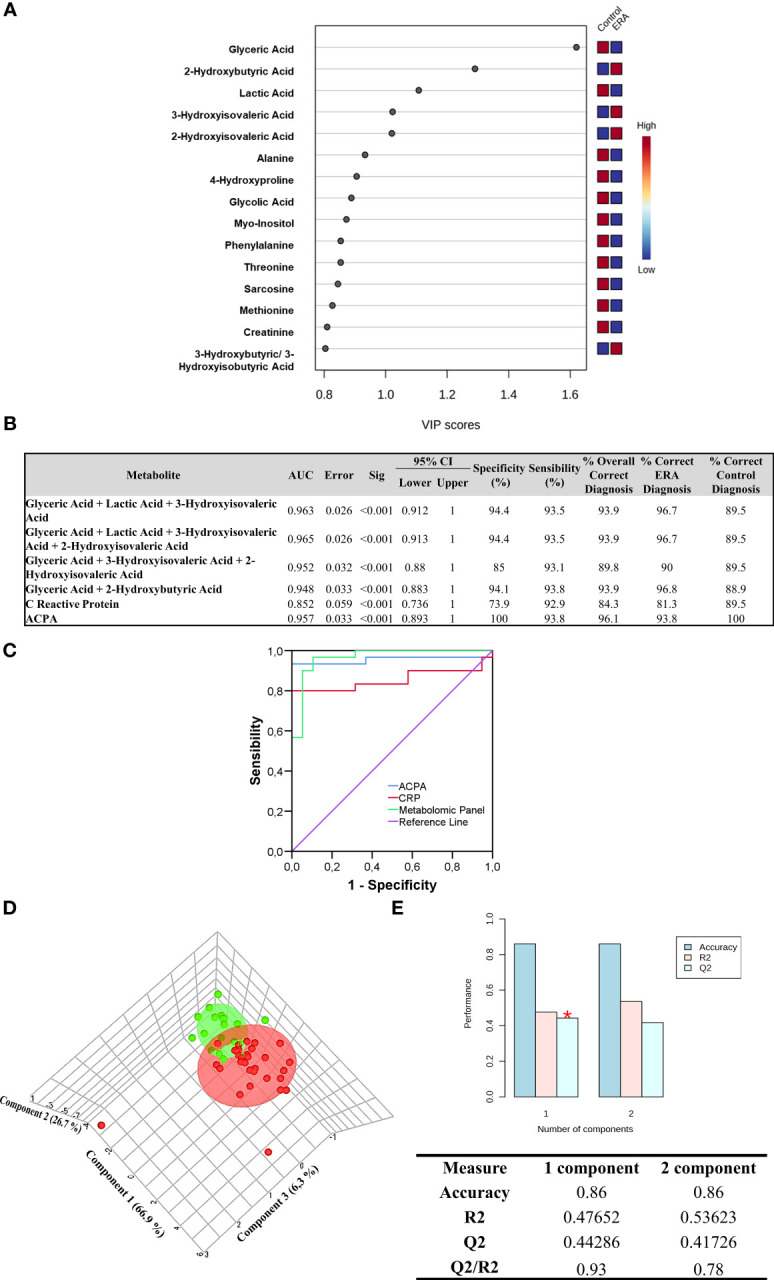
Metabolite ERA panel design. **(A)** Variable importance in projection (VIP) scores of the 15 selected variables are shown in the model (Glyceric Acid, 2-Hydroxybutyric Acid, Lactic Acid, 3-Hydroxyisovaleric Acid, 2-Hydroxyisovaleric Acid, Alanine, 4-Hydroxyproline, Glycolic Acid, Myo-Inositol, Phenylalanine, Threonine, Sarcosine, Methionine, Creatinine and 3-Hydroxybutyric/3-Hydroxyisobutyric Acid). Variables with scores close to or greater than 1 were considered important in the model. The column to the right displays a heatmap showing the abundance of the top 15 metabolites based on VIP scores of patients with ERA versus healthy controls. **(B)** Metabolite-based best models as diagnostic classifiers for ERA. **(C)** Receiver operating characteristic (ROC) curve values showing predictive efficiency for distinguishing ERA from healthy controls. Percentage of correct diagnostic values was obtained by multivariate models AUC, area under the curve; 95% CI (confidence interval). **(D)** Partial least squares discriminant analysis (PLS-DA) model to evaluate the potential of the three metabolites to discriminate between ERA and controls in 3D. **(E)**Values of the classification performance assessed by accuracy, goodness of fit (R2), and predictive ability (Q2) for the top three components. Three components best classify the model shown with the red asterisk using leave-one-out cross-validation.

We next performed a VIP analysis to examine the contribution of the 15 metabolites in determining the presence of ERA. We found that 5/15 metabolites had a VIP score ≥1 (glyceric acid, lactic acid, 2-hydroxybutyric acid, 3-hydroxysovaleric acid, and 2-hydroxyvaleric acid) and were considered important in the model for determining ERA onset (**Figure 2A**).

To evaluate the usefulness of the five metabolites as potential diagnostic biomarkers of ERA in serum, we next performed logistic regression analysis and generated ROC curves. **Supplementary Material**, **Table S2** lists the different combinations performed with the five selected metabolites. The results showed that the area under the curve (AUC) of each individual variable, such as lactic acid, 3-hydroxybutyric acid, 3-hydroxyisovaleric acid, and 2-hydroxyisovaleric acid, was below 0.8, and only glyceric acid showed an AUC above 0.8. Notably, the presence of glyceric acid outperformed the other individual variables, alone or in combination. We then generated a multivariate regression model combining each potential metabolite to test which combination was better for ERA diagnosis. Results showed that two models had the highest AUC, specificity, and sensitivity values. The first panel comprised glyceric acid, lactic acid, and 3-hydroxyisovaleric acid, with an AUC of 0.963 and with 94.4% specificity and 93.5% sensitivity, and could correctly classify 96.7% of patients with ERA. The second panel comprised glyceric acid, lactic acid, 3-hydroxyisovaleric acid, and 2-hydroxyisovaleric acid, with an AUC of 0.965 and with 94.4% specificity and 93.5% sensitivity, and could also correctly classify 96.7% of patients with ERA **(** **Figure 2B**). Accordingly, the best metabolite combination panel for identifying ERA was the first panel, as it comprised only three metabolites. Notably, comparison of the ROC curves of ACPA, CRP, and the selected metabolomic panel (glyceric acid, lactic acid, and 3-hydroxyisovaleric acid) for ERA diagnosis revealed that the latter outperformed classical ACPA biomarker diagnosis by 2.9% and CRP by 15.4%, thus improving the preclinical detection of ERA (**Figures 2B, C**).

The selected metabolomic panel for ERA diagnosis (glyceric acid, lactic acid, 3-hydroxyisovaleric acid) was then back-evaluated with PLS-DA to test the strength of the model. The results showed that subjects were clearly segregated into two differentiated groups: control and ERA (**Figure 2D**). The PLS-DA model overfitting, measured as the Q2/R2 ratio, was 0.93, indicating that the model has strong accuracy and clearly distinguishes patients with ERA from healthy controls (**Figure 2E**).

### 3.3 Metabolite functional enrichment analysis

**Figure 3:**
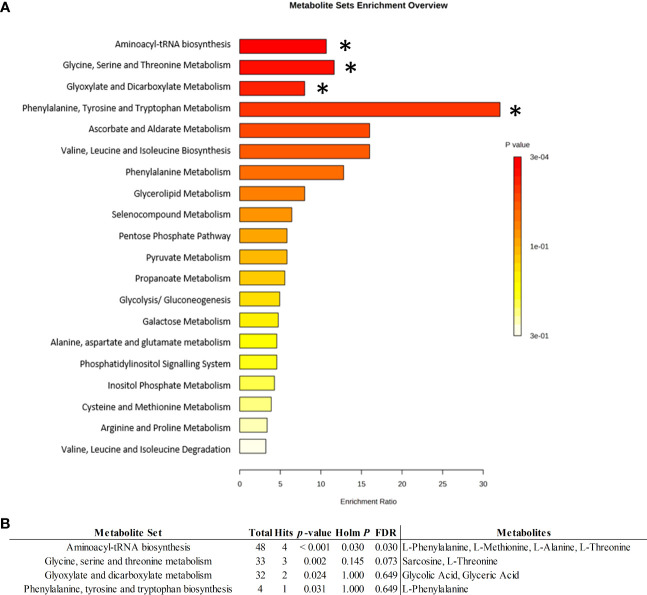
Metabolic set enrichment analysis. **(A)** Metabolite set enrichment overview from quantitative enrichment analysis by Kyoto Encyclopedia of Genes and Genomes (KEGG) metabolite set library, showing corresponding fold enrichments and computed p-values (*p<0.005). Bar colors are based on p-values (lower p-values correspond to darker red), while bar lengths are based on the fold enrichment. **(B)** Identified ERA discriminant metabolites allocated to related pathways. Total: total metabolites allocated in the KEGG pathway; Hits: number of ERA-altered metabolites marching in the pathway; HolmP: Bonferroni Holm<-p. Adjust method; FDR, False discovery Rate.

Although not all 15 deregulated metabolites were selected as a part of the biomarker panel, they might all function at the onset of RA by being higher or lower in the disease environment. We, thus, performed functional analyses with all 15 metabolites using the KEGG metabolite library to have a better understanding of the pathophysiology of ERA. The results showed that four pathways were significantly disturbed: aminoacyl-tRNA biosynthesis; glycine, serine, and threonine metabolism; glyoxylate and dicarboxylate metabolism; and phenylalanine, tyrosine, and tryptophan biosynthesis (**Figure 3A**). Pathways are shown in order of decreasing significance from top to bottom based on *p*-values and false discovery rate values in **Figure 3B**. With respect to aminoacyl-tRNA biosynthesis, 4/48 metabolites incorporated in the KEGG library for this pathway were found to match in the pathway: L-phenylalanine, L-methionine, L-alanine, and L-threonine. All were found to be decreased in the serum of patients with ERA. For the glycine, serine, and threonine metabolism pathways, 3/33 metabolites were identified: sarcosine, L-threonine, and glyceric acid. For glyoxylate and dicarboxylate metabolism, 2/32 metabolites were identified: glycolic acid and glyceric acid. Finally, in the phenylalanine, tyrosine, and tryptophan biosynthesis pathway, 1 of 4 metabolites was identified: L-phenylalanine (**Figure 3B**).

## 4 Discussion

The metabolic changes that occur in patients with ERA are complex and interconnected, involving several pathways and mechanisms (4). Although the specific metabolic alterations may differ between patients and disease stages, the identification of shared metabolic networks might provide a tool for disease diagnosis that yields insights into disease-related mechanisms. Here we have identified a metabolic signature to aid in the early diagnosis of RA. Our findings reveal that 15 of 81 detected serum metabolites showed differences in concentration between patients with ERA and healthy controls.

Decision model analysis incorporating the 15 dysregulated metabolites revealed that the best discriminatory metabolites for ERA diagnosis were glyceric acid, 2-hydroxybutyric acid, lactic acid, 3-hydroxyisovaleric acid, and 2-hydroxyisovaleric acid. Further regression analysis using combinations of these five metabolites showed that a panel consisting of glyceric acid, lactic acid, and 3-hydroxyisovaleric acid was superior for ERA patient identification with 96.7% accuracy, outperforming the classical ACPA marker by 2.9% and the CRP inflammatory marker by 15.4%.

Pathway analysis of the dysregulated metabolites uncovered amino acid metabolism as one of the most affected pathways in ERA; specifically, aminoacyl t-RNA biosynthesis, which plays an important role in protein synthesis by binding amino acids to their tRNAs. This route has been previously described as related to immune responses as well as being dysregulated in autoimmune diseases such as RA (12). Indeed, a study attempting to predict synovial gene expression by analyzing serum metabolic profiles of patients with RA on methotrexate concluded that serine/glycine/phenylalanine metabolism and the biosynthesis of aminoacyl-tRNAs were related to TNF-α/CD3E and B-cell/plasma signatures (13), pointing to the involvement of these pathways in the regulation of lymphocytes in the rheumatoid synovium.

Several metabolomics studies using biofluids obtained from patients with established RA have reported a decrease in amino acid levels together with a decrease in glucose and an increase in fatty acid metabolites and serum cholesterol (14, 15). For instance, Zhou et al. (16) reported that the altered metabolites in serum from patients with established RA feed into pathways such as glycolysis, fatty acid, and amino acid metabolism, tricarboxylic acid cycle, and the urea cycle.

Glyceric acid was one of the three signature metabolites in our diagnostic panel that was found at lower levels in patients with ERA than in healthy controls. Glyceric acid is a product of glycerol oxidation; in turn, glycerol can be used to synthesize triacylglycerols through glycerolipid metabolism, which is increased in patients with pre-clinical and ERA (17). Conversely, other authors (18) found higher glyceric acid levels in serum metabolomic studies when comparing patients with RA and healthy controls. This discrepancy with our findings could be due to the different cohort composition in the aforementioned study, which included patients with established RA and patients who were being treated with disease-modifying antirheumatic drugs and corticosteroids.

The second metabolite constituting our ERA panel was lactic acid, which was increased in the serum of patients with ERA when compared with control counterparts, albeit not reaching significance, likely due to the small sample number. Lactic acid is an essential organic acid in cellular metabolism that also contributes to the progression of RA from the first phases of inflammation to the last phases of bone destruction (19). Lactate is mainly produced in the cytoplasm under conditions of hypoxia or high-throughput glycolysis in rapidly proliferating cells – two processes that occur in RA – which can explain the increased concentration found in the synovial fluid of patients with RA (20, 21). The inflamed joint has been recognized as a site with low levels of glucose and high amounts of lactate, which is in part responsible for the acidic environment of RA synovitis (14).

Our proposed panel model also included the metabolite 3-hydroxyisovaleric acid, a by-product of the leucine degradation pathway, which we found in significantly higher amounts in patients with ERA. A previous metabolomics study using urine samples from patients with immune-mediated inflammatory diseases found lower levels of 3-hydroxyisovaleric acid in the disease cohort than in controls (22), which contrasts with our findings. However, the cohorts differed between this study and ours, as did the sample type and detection method. While we found lower levels of serum 3-hydroxyisovaleric acid, the serum levels of leucine were not significantly different between ERA and control cohorts in our study, likely because the differences in the levels of 3-hydroxyisovaleric acid in the serum of patients with ERA compared with controls were not sufficiently high to reflect leucine changes at an early stage of the RA pathology. In line with this finding, different levels of leucine have been reported according to RA disease progression and are associated with other factors such as age, sex, and diet (23). Leucine levels have also been linked to muscle protein breakdown in response to energy expenditure and inflammation (24).

We are aware that one limitation in our study is the lack of validation in a different ERA cohort; however, patients with RA naïve to treatment are not easy to recruit. Moreover, further research will be needed to establish the clinical utility of our proposed panel to standardize their use for routine practice.

In conclusion, a serum metabolite signature panel composed of glyceric acid, lactic acid, and 3-hydroxyisovaleric acid was the best combination for ERA diagnosis, outperforming classical clinical diagnostic markers such as ACPA. Notably, the application of metabolomics can help to better elucidate pathways involved in ERA, which might guide the development of novel approaches for its diagnosis and/or treatment. Despite these advantages, however, it is important to note that metabolomic panels are still an emerging technology in the clinical setting. As medical research advances, the integration of metabolomic panels and other advanced technologies into clinical practice may revolutionize ERA diagnosis, treatment, and management, ultimately improving patient outcomes.

## Data availability statement

The original contributions presented in the study are publicly available. This data can be found here: https://doi.org/10.5281/zenodo.8131016.

## Ethics statement

The studies involving humans were approved by Pere Virgili Research Institute Ethic Committee (Ref. CEIM: 047/2021).https://www.iispv.cat/en/ceim/comite-etic-dinvestigacio-amb-medicamets-ceim-institut-dinvestigacio-sanitaria-pere-virgili/. The studies were conducted in accordance with the local legislation and institutional requirements. The participants provided their written informed consent to participate in this study.

## Author contributions

All authors were involved in drafting the article or revising it critically for important intellectual content, and all authors approved the final version to be published. SR-M, AA-C and MRC. had full access to all the data in the study and take responsibility for the integrity of the data and the accuracy of the data analysis. Study conception and design: SR-M, AA-C, RF-G and MRC. Acquisition of data: SC-O, MJP-E, NdC-P. Analysis of the data: MM-H, VA-G and interpretation of data. AA-C, SR-M, RF-G and MRC.
