# Supplementary material for: A serum metabolic biomarker panel for early rheumatoid arthritis
Source: Front Immunol. 2023 Sep 1;14:1253913. doi: 10.3389/fimmu.2023.1253913 (PMC10502709; doi:10.3389/fimmu.2023.1253913)
Supplement: Supplementary file 1 [file Table_1.pdf]

Table S1. Metabolites detected in serum of ERA and control patients.

**Metabolites (Relative Units)**	**CONTROL**	**ERA**	***p-value***
	**Median (IQR)**	**Median (IQR)**	
**Pyruvic acid**	3.044 (2.13, 4.07)	3.14 (2.22, 4.05)	0.922
**Lactic Acid**	25.90 (24.16, 28.24)	28.46 (24.04, 31.44)	0.070

**2-Hydroxyisobutyric Acid**	0.24 (0.19, 0.27)	0.24 (0.20, 0.32)	0.436
**Glycolic Acid**	0.09 (0.08, 0.11)	0.08 (0.07, 0.10)	0.050
**Alanine**	20.64 (18.70,21.22)	18.71 (17.05, 20.83)	0.073
**2-Hydroxybutyric Acid**	2.69 (1.59, 2.99)	3.27 (2.30, 4.77)	0.024
**Sarcosine**	0.06 (0.05, 0.06)	0.05 (0.05, 0.05)	0.017
**3-Methyl-2-Oxobutyric Acid (Alphaketoisovaleric Acid)**	0.09 (0.08, 0.10)	0.09 (0.07, 0.12)	0.815
**Oxalic Acid**	11.80 (9.46, 13.68)	12.32 (7.58, 16.27)	0.682
**3-Hydroxybutyric Acid/ 3-Hydroxyisobutyric Acid**	1.09 (0.57, 1.56)	1.58 (1.02, 3.71)	0,061
**2-Hydroxyisovaleric Acid**	0.70 (0.50, 1.04)	0.85 (0.72, 1.25)	0.073
**2-Keto-3-Methylvaleric Acid**	0.19 (0.15, 0.24)	0.19 (0.17, 0.23)	0.907
**3-Hydroxyisovaleric Acid**	0.11 (0.09, 0.14)	0.14 (0.11, 0.16)	0.017
**2-Ketoisocaproic Acid**	0.37 (0.33, 0.49)	0.38 (0.30, 0.46)	0.267
**Valine**	14.44 (13.58,15.16)	14.27 (13.39, 15.93)	0.846

**Ethanolamine**	0.29 (0.24, 0.31)	0.26 (0.22, 0.29)	0.139
**Leucine**	9.09 (8.46, 10.46)	8.91 (8.13, 9.82)	0.471
**Phosphoric Acid**	46.28 (38.47,50.98)	45.06 (39.64, 52.03)	0.876
**Isoleucine**	4.46 (4.14, 5.17)	4.51 (3.94, 4.94)	0,785
**Proline**	42.58 (34.15,48.61)	36.87 (31.10, 46.06)	0,284
**Acetylglycine**	0.02 (0.01, 0.02)	0.02 (0.02, 0.02)	0.755
**Glycine**	4.72 (4.43, 4.95)	4.87 (4.48, 5.03)	0.242
**Succinic Acid**	0.06 (0.05, 0.07)	0.06 (0.05, 0.06)	0.117
**Glyceric Acid**	0.88 (0.66, 1.05)	0.45 (0.28, 0.54)	<0.001
**Serine**	9.70 (8.62, 10.33)	8.85 (7.80, 10.32)	0.330
**Threonine**	9.56 (8.01, 10.06)	8.25(6.99, 9.40)	0.059
**Malic Acid**	0.05 (0.04, 0.06)	0.04 (0.04, 0.05)	0. 223
**D-Threitol**	0.00 (0.00, 0.01)	0.00 (0.00, 0.01)	0.375
**Methionine**	2.18 (1.84, 2.45)	1.89 (1.64, 1.99)	0.008

**Oxoproline**	61.24 (52.46,68.86)	58.71 (47.21, 70.04)	0.483
**Aspartic**	0.38 (0.30, 0.42)	0.34 (0.27, 0.45)	0.391
**Iminodiacetic Acid**	0.10 (0.08, 0.14)	0.09 (0.07, 0.11)	0.192
**4-Hydroxyproline**	3.76 (2.62, 4.78)	2.83 (2.47, 3.91)	0.067
**Threonic Acid**	2.26 (1.83, 2.50)	2.01 (1.75, 2.35)	0.149
**Erythronic Acid**	0.03 (0.03, 0.04)	0.03 (0.02, 0.04)	0.424
**Creatinine**	0.39 (0.34, 0.48)	0.36 (0.31, 0.43)	0.039
**DL-2-Hydroxyglutaric Acid**	0.03 (0.03, 0.04)	0.03 (0.03, 0.04)	0.718
**A-Ketoglutaric Acid**	0.84 (0.64, 1.02)	0.77 (0.59, 0.98)	0.391
**Glutamic Acid**	5.35 (4.58, 8.03)	5.61 (4.23, 7.03)	0.546
**4-Hydroxybenzoic Acid**	0.12 (0.11, 0.12)	0.11 (0.11, 0.12)	0.471
**Phenylalanine**	7.84 (7.51, 8.13)	7.18 (6.62, 7.62)	0.004
**D-Xylose**	0.01 (0.01, 0.01)	0.01 (0.01, 0.01)	0.297
**Taurine**	2.03 (1.06, 3.40)	2.88 (1.70, 5.79)	0.098

**D-Arabinose**	0.01 (0.01, 0.01)	0.01 (0.01, 0.01)	0.055
**d-Xylulose**	0.00 (0.00, 0.00)	0.00 (0.00, 0.00)	0.791
**D-Xylitol**	0.00 (0.00, 0.00)	0.00 (0.00, 0.00)	0.157
**D-Arabitol**	0.01 (0.01, 0.01)	0.01 (0.01, 0.01)	0.441
**Glycerol-1-Phosphate**	0.30 (0.22, 0.33)	0.23 (0.18, 0.32)	0.220
**Glutamine**	31.17 (29.07,35.73)	30.94 (26.73, 38.10)	0.938
**Xylonic Acid**	0.02 (0.02, 0.02)	0.02 (0.01, 0.02)	0.325
**Ribonic Acid**	0.00 (0.00, 0.01)	0.00 (0.00, 0.01)	0.044
**Hypoxanthine**	0.66 (0.52, 0.78)	0.74 (0.55, 0.92)	0.231
**Ornithine**	15.40 (12.51, 17.91)	16.21 (13.95, 18.85)	0.559
**Citric Acid**	73.73 (44.59,88.99)	64.03 (45.33, 83.61)	0,755
**Hippuric Acid**	2.10 (1.21, 4.75)	1.30 (0.85, 3.09)	0.098
**Tetradecanoic Acid**	0.94 (0.66, 1.25)	0.82 (0.66, 1.06)	0.330
**4-Hydroxyphenyllactic Acid**	1.85 (1.26, 2.06)	1.69 (1.03, 1.95)	0.559

**D-Fructose**	0.05 (0.04, 0.07)	0.06 (0.36, 0.09)	0.316
**Galactose**	8.87 (7.55, 11.28)	9.76 (8.06, 11.62)	0.235
**D-Mannitol**	0.02 (0.01, 0.05)	0.02 (0.02, 0.06)	0.592
**D-Sorbitol**	0.14 (0.06, 0.20)	0.15 (0.08, 0.30)	0.365
**D-Mannonic Acid**	0.06 (0.04, 0.10)	0.07 (0.04, 0.13)	0.899
**Galacturonic Acid**	0.04 (0.03, 0.04)	0.04 (0.03, 0.05)	0.755
**D-Galactitol**	0.03 (0.03, 0.04)	0.03 (0.03, 0.04)	0.740
**D-Gluconic Acid**	0.01 (0.01, 0.01)	0.01 (0.01, 0.01)	0.853
**Galactonic Acid**	0.00 (0.00, 0.00)	0.00 (0.00, 0.00)	0.689
**Saccharic Acid**	0.01 (0.01, 0.02)	0.02 (0.02, 0.02)	0.447
**Indole-3-Propanoic Acid**	0.13 (0.07, 0.21)	0.11 (0.07, 0.21)	0.697
**Myo-Inositol**	0.67 (0.59, 0.83)	0.57 (0.50, 0.66)	0.029
**Uric Acid**	30.41 (21.31, 36.84)	29.52 (19.66, 34.49)	0.350
**Sedoheptulose-1**	0.02 (0.02, 0.03)	0.02 (0.02, 0.03)	0.592

**Sedoheptulose-2**	0.03 (0.02, 0.03)	0.03 (0.02, 0.03)	0.992
**Indolelactic Acid**	0.13 (0.10, 1.13)	0.11 (0.09, 0.15)	0.267
**Linoleic Acid**	2.63 (1.13, 3.68)	2.06 (1.40, 3.36)	0.922
**Oleic Acid**	13.39 (10.46,26.83)	17.51 (8.53, 25.02)	0.892
**Stearic Acid**	63.26 (57.95,72.43)	64.74 (58.60, 72.69)	0,559
**Glucose 6-Phosphate**	0.01 (0.01, 0.01)	0.01 (0.01, 0.01)	0.002
**D-Sucrose**	0.00 (0.00, 0.01)	0.00 (0.00, 0.01)	0.654
**Maltose-iso-1**	0.01 (0.01, 0.02)	0.02 (0.01, 0.03)	0.173
**Maltose-iso-2**	0.00 (0.00, 0.00)	0.00 (0.00, 0.01)	0.104
**A-Tocopherol**	0.88 (0.48, 1.49)	0.79 (0.63, 1.30)	0.785
