# Supplementary material for: A serum metabolic biomarker panel for early rheumatoid arthritis
Source: Front Immunol. 2023 Sep 1;14:1253913. doi: 10.3389/fimmu.2023.1253913 (PMC10502709; doi:10.3389/fimmu.2023.1253913)
Supplement: Supplementary file 2 [file Table_2.pdf]

frontiers

Table S2 . Metabolites based models as diagnostic classifiers for ERA with the 5 selected variables.

Metabolites	AUC	Error	Sig	95% CI		Specificity (%)	Sensibility (%)	% Overall Correct Diagnosis	% Correct ERA Diagnosis	% Correct Control Diagnosis
				Lower	Upper					
**Glyceric Acid + Lactic Acid + 3-Hydroxyisovaleric Acid**	0.963	0.026	<0.001	0.912	1	94.4	93.5	93.9	96.7	89.5
**Glyceric Acid + Lactic Acid + 3-Hydroxyisovaleric Acid + 2-Hydroxyisovaleric Acid**	0.965	0.026	<0.001	0.913	1	94.4	93.5	93.9	96.7	89.5
**Glyceric Acid + 3-Hydroxyisovaleric Acid + 2-Hydroxyisovaleric Acid**	0.952	0.032	<0.001	0.88	1	85	93.1	89.8	90	89.5
**Glyceric Acid + 2-Hydroxybutyric Acid**	0.948	0.033	<0.001	0.883	1	94.1	93.8	93.9	96.8	88.9

**Glyceric Acid + 2-Hydroxybutyric Acid + Lactic Acid**	0.95	0.033	<0.001	0.886	1	94.1	93.8	93.9	96.8	88.9
**Glyceric Acid + 2-Hydroxybutyric Acid + Lactic Acid + 2-Hydroxyisovaleric Acid**	0.957	0.028	<0.001	0.903	1	94.1	93.8	93.9	96.8	88.9
**Glyceric Acid + 2-Hydroxybutyric Acid + 2-Hydroxyisovaleric Acid**	0.957	0.028	<0.001	0.903	1	94.1	93.8	93.9	96.8	88.9
**Glyceric Acid + 2-Hydroxybutyric Acid + Lactic Acid + 3-Hydroxyisovaleric Acid**	0.965	0.025	<0.001	0.915	1	94.1	93.5	93.8	96.7	88.9
**Glyceric Acid + 2-Hydroxybutyric Acid + Lactic Acid + 3-Hydroxyisovaleric Acid + 2-Hydroxyisovaleric Acid**	0.965	0.025	<0.001	0.915	1	94.1	93.5	93.8	96.7	88.9
**Glyceric Acid + 2-Hydroxybutyric Acid + 3-Hydroxyisovaleric Acid**	0.965	0.025	<0.001	0.915	1	94.1	93.5	93.8	96.7	88.9
**Glyceric Acid + 2-Hydroxybutyric Acid + 3-Hydroxyisovaleric Acid + 2-Hydroxyisovaleric**	0.965	0.025	<0.001	0.915	1	94.1	93.5	93.8	96.7	88.9
**Glyceric Acid**	0.944	0.033	<0.001	0.88	1	94.1	90.9	92	96.8	84.2
**Glyceric Acid + Lactic Acid**	0.956	0.028	<0.001	0.9	1	88.9	90.6	90	93.5	84.2
**Glyceric Acid + Lactic Acid + 2-Hydroxyisovaleric Acid**	0.956	0.031	<0.001	0.896	1	88.9	90.6	90	93.5	84.2
**Glyceric Acid + 2-Hydroxyisovaleric Acid**	0.956	0.031	<0.001	0.896	1	84.2	90.3	88	90.3	84.2
**Glyceric Acid + 3-Hydroxyisovaleric Acid**	0.957	0.028	<0.001	0.902	1	84.2	90	87.8	90	84.2
**2-Hydroxybutyric Acid + 3-Hydroxyisovaleric Acid + 2-Hydroxyisovaleric**	0.789	0.065	0.001	0.661	0.916	61.1	77.4	71.4	77.4	61.1
**2-Hydroxybutyric Acid + Lactic Acid + 3-Hydroxyisovaleric Acid**	0.791	0.065	0.001	0.663	0.919	62.5	75.8	71.4	80.6	55.6

**2-Hydroxybutyric Acid + Lactic Acid + 3-Hydroxyisovaleric Acid + 2-Hydroxyisovaleric Acid**	0.789	0.066	0.001	0.66	0.917	62.5	75.8	71.4	80.6	55.6
**2-Hydroxybutyric Acid + 3-Hydroxyisovaleric Acid**	0.78	0.066	0.001	0.649	0.91	58.8	75	69.4	77.4	55.6
**2-Hydroxybutyric Acid + Lactic Acid + 2-Hydroxyisovaleric Acid**	0.757	0.068	0.003	0.623	0.891	52.9	72.7	66	75	50
**2-Hydroxybutyric Acid + 2-Hydroxyisovaleric Acid**	0.752	0.069	0.004	0.617	0.887	52.9	72.7	66	75	50
**3-Hydroxyisovaleric Acid + 2-Hydroxyisovaleric Acid**	0.734	0.073	0.005	0.6	0.885	64.3	72.2	70	83.9	47.4
**2-Hydroxybutyric Acid**	0.735	0.071	0.007	0.596	0.875	61.5	73	70	84.4	44.4
**2-Hydroxybutyric Acid + Lactic Acid**	0.735	0.071	0.007	0.596	0.875	57.1	72.2	68	81.3	44.4
**3-Hydroxyisovaleric Acid**	0.739	0.073	0.006	0.595	0.883	66.7	71.1	70	87.1	42.1
**Lactic Acid + 3-Hydroxyisovaleric Acid**	0.715	0.076	0.013	0.566	0.863	57.1	69.4	66	80.6	42.1
**Lactic Acid + 3-Hydroxyisovaleric Acid + 2-Hydroxyisovaleric Acid**	0.733	0.074	0.007	0.588	0.879	57.1	69.4	66	80.6	42.1
**Lactic Acid + 2-Hydroxyisovaleric Acid**	0.693	0.079	0.027	0.537	0.848	58.3	69.2	66.7	84.4	36.8
**2-Hydroxyisovaleric Acid**	0.674	0.084	0.045	0.509	0.839	54.5	67.5	64.7	84.4	31.6
**Lactic Acid**	0.639	0.079	0.11	0.484	0.794	44.4	64.3	60.8	84.4	21.1

Receiver operating characteristic (ROC) curve values showing the predictive efficiency for ERA diagnosis Percentage of correct diagnostic values was obtained by multivariate models (backward stepwise, conditional method). AUC, area under the curve; 95% CI (confidence interval). The bolded words represent the ROC Models.
