# Supplementary material for: A serum metabolic biomarker panel for early rheumatoid arthritis
Source: Front Immunol. 2023 Sep 1;14:1253913. doi: 10.3389/fimmu.2023.1253913 (PMC10502709; doi:10.3389/fimmu.2023.1253913)

Figure S1.

(A) Partial least squares discriminant analysis (PLS-DA) model to evaluate the potential of the 15 altered metabolites to discriminate between ERA and controls in 3-dimension.

(B) Prediction accuracy of PLS-DA, showing values of R2 and Q2.

(C) Heat map of the clustering analysis with the 15 altered metabolites. Metabolite intensities are displayed as colours ranging from red-grading (more abundant metabolites) to blue-grading (less abundant metabolites).

A)

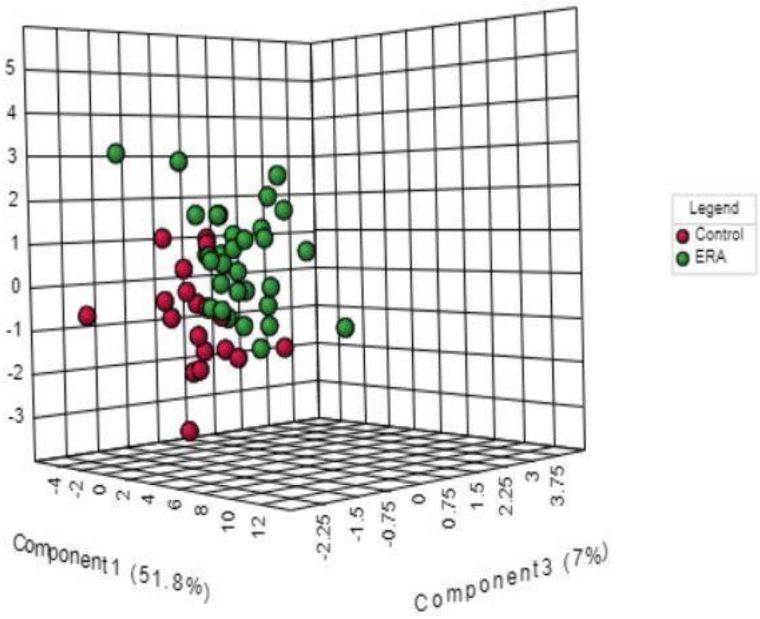

B)

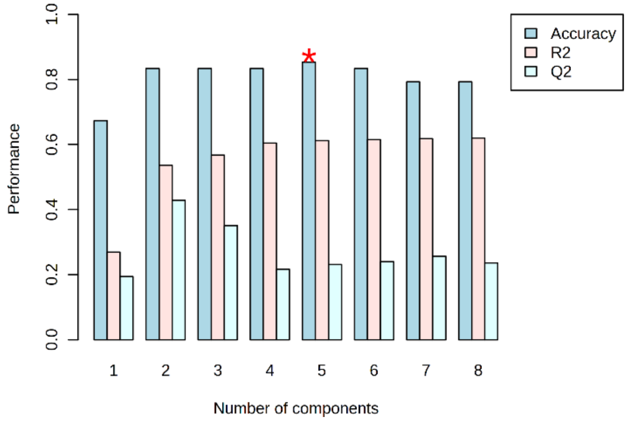

Measure	1 comps	2 comps	3 comps	4 comps	5 comps	6 comps	7 comps	8 comps
Accuracy	0.67	0.83	0.83	0.83	0.85	0.83	0.79	0.79
R2	0.27	0.54	0.57	0.60	0.61	0.61	0.62	0.62
Q2	0.19	0.43	0.35	0.22	0.23	0.24	0.26	0.23
Q2/R2	0.72	0.80	0.62	0.36	0.38	0.39	0.41	0.38

C)

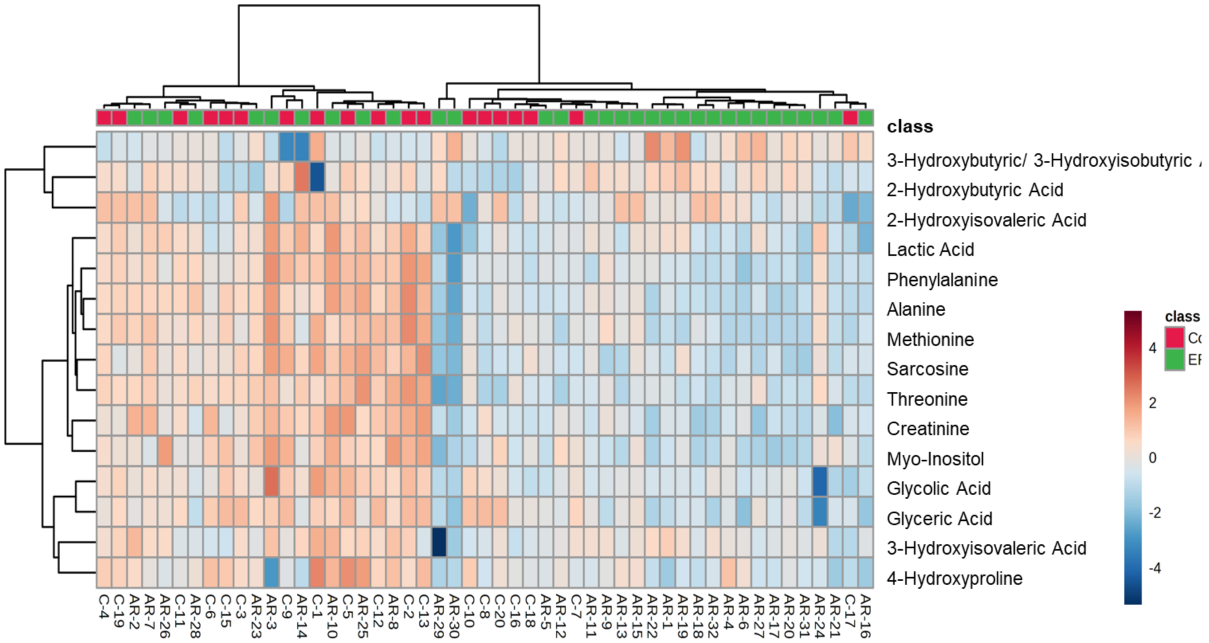

Supplement: Supplementary file 3 [file Image_1.pdf]
